# Paired Multiple-Choice Questions Reveal Students’ Incomplete Statistical Thinking about Variation during Data Analysis

**DOI:** 10.1128/jmbe.00112-21

**Published:** 2021-05-31

**Authors:** Jenna Hicks, Jessica Dewey, Michael Abebe, Yaniv Brandvain, Anita Schuchardt

**Affiliations:** a Department of Biology Teaching and Learning, University of Minnesota, Minneapolis, Minnesota, USA; b STEM Education Center, University of Minnesota, St. Paul, Minnesota, USA; c Department of Ecology, Evolution, and Behavior, University of Minnesota, St. Paul, Minnesota, USA; d Department of Plant and Microbial Biology, University of Minnesota, St. Paul, Minnesota, USA

**Keywords:** education, assessments, statistics, undergraduate, variation

## Abstract

Biologists consider variability during biological investigations. A robust quantitative understanding of variability is particularly important during data analysis, where statistics are used to quantify variation and draw conclusions about phenomena while accounting for variation. Many students struggle to correctly apply a quantitative understanding of variation to statistically analyze data. We present quantitative and qualitative analyses of introductory biology students’ responses on two pairs of multiple-choice questions querying two concepts related to the quantitative analysis of variation. More students correctly identify a mathematical expression of variation than correctly interpret it. Many students correctly interpret a nonsignificant *p*-value in the context of a very small sample size, but fewer students do so in the context of a large sample size. These results imply that many students have an incomplete quantitative understanding of variation. These findings suggest that instruction focusing on conceptual understanding, not procedural problem solving, may elevate students’ quantitative understanding of variation.

## INTRODUCTION

Biology and the study of biological phenomena are inherently variable. Biological investigations have to consider both variability, the propensity for change of a characteristic or entity under study, and variation, the measuring of that characteristic (1). When experts are planning a biological investigation, they consider variability throughout all phases of the investigation from both a conceptual perspective (e.g., “What sources of variability do I anticipate?”) and a quantitative perspective (e.g., “How large of an effect will that source of variability have on the measured outcome?”) (2, 3). The ability to use quantitative skills to reason about biological phenomena (e.g., variability/variation) and the ability to conduct a biological investigation have been prioritized as core competencies for students in biology (4, 5). However, both of these competencies can be challenging for students (6, 7). Applying quantitative thinking to consider and account for variability during a biological investigation is even more challenging: students must apply their quantitative thinking skills to reason about an abstract concept (variability) in the context of a multistep, complex process (a biological investigation) (6–9). In order to design instruction that helps students overcome these challenges, educators must first characterize students’ ideas to identify specific areas of difficulty. This study presents analyses of data from introductory undergraduate biology students’ responses on two pairs of multiple-choice questions used to characterize students’ quantitative understanding of variation in the context of a biological investigation.

### Students struggle to quantitatively consider variation when carrying out investigations

When conducting a biological investigation, researchers identify a research question, plan an experiment to address the question, collect data, analyze the data, and ultimately use these analyses to make conclusions about the biological phenomenon under study (2, 3). Biologists must quantitatively consider variation throughout the investigation process so that the study is designed to allow relevant data to be collected, permitting appropriate analyses (2, 10). In the initial design phases, researchers must consider (i) the number of sources of variability that might impact the measured outcome (e.g., genetic variability, environmental variability, or variability due to measurement inaccuracies), (ii) the magnitude of the anticipated effect of said sources of variability on the measured outcome, and (iii) how large a sample size is needed to achieve satisfactory statistical power (3, 11). For example, a researcher studying the effects of a drug compound on the expression level of a hypothetical ProteinX in mice must consider normal variability in the level of ProteinX between mice due to genetic variability or environmental factors, variability introduced by the researcher during drug administration and collection of samples, and the variation that can arise due to the (im)precision of the tools used to measure ProteinX. If the drug is expected to have only a modest effect on ProteinX expression, the researcher would likely conduct a study with a larger number of mice in an effort to gain greater statistical power. In the data analysis phase, researchers often quantify the amount of variation present in the measured outcome, and use statistical tests to determine the probabilistic significance of their observed results (12, 13).

Applying quantitative thinking skills to conduct statistical analyses of data are particularly difficult for many students (14–18). Some students struggle to understand how descriptive statistics are used to represent the distribution of data during the initial steps in the data analysis process (6, 19–21). For example, nearly half of students in an introductory biology course did not correctly calculate and represent the mean of a data set on a graph at the beginning of the course, instead choosing to plot the values of individual replicates or the sum of the replicate values (6). Student challenges with connecting descriptive statistics with the concepts that they represent may be due to difficulties with making sense of mathematical representations in applied contexts (22–24) and/or reliance on rule-based or procedural approaches to problem solving (25–29).

Many studies report students’ and experts’ difficulty with understanding hypothesis testing (such as *t* tests) and interpreting *p*-values (14, 17, 18, 30–35). However, many of these studies of students’ understanding were conducted in the context of statistics courses, not biology courses (14, 16–18). A few studies have focused on students’ ideas about applying and interpreting statistical tests in the context of biology courses (6, 30, 31). Some of these studies indicate that even when statistics instruction is embedded within biology courses, incorrect ideas about *p*-values and statistical tests can be resistant to change (30, 31). This is perhaps not very surprising, since incorrect interpretations of *p*-values are pervasive, even with practicing scientists (34–36). Misinterpretations of *p*-values can result in underconfident interpretations of a result (e.g., falsely concluding that an experimental manipulation did not have an effect because *p* = 0.055, despite a large effect size) or overconfident interpretations of a result (e.g., falsely concluding that an experimental manipulation did have an effect because *p* = 0.045, despite a small effect size with no practical importance). Using *p*-values as the sole indicator of experimental effect can lead to many incorrect ideas, including equating statistically significant *p*-values with biological significance, conflating *p*-values and effect sizes, and equating “failing to reject the null hypothesis” and proving that the null hypothesis is true. (15, 36, 37). It is important to characterize specific aspects of students’ understanding of these difficult topics (e.g., *p*-values and statistical tests) so instruction can be designed to elevate students’ understanding.

### Multiple-choice questions can be used to easily characterize and assess students’ ideas

Characterizing students’ ideas can provide useful insights about curricular needs. However, obtaining specific information about students’ understanding of complex topics can be difficult for instructors who have limited time. Recent trends in assessment of scientific (including biological) content knowledge have suggested that open-ended questions and tasks afford more opportunities to obtain a comprehensive representation of students’ ideas (38–40). However, these open-ended question formats require more time for students to complete (41), and more time and effort for instructors to evaluate (42, 43). Multiple-choice questions can be administered and analyzed quickly using inexpensive software or by hand. In addition, studies indicate that multiple-choice and free response question formats can be equivalent in their ability to elicit information about respondents’ ideas, discriminate between novices and advanced learners, and assess higher-order thinking (44–46).

Closed-ended questions (like multiple-choice or multiple-true/false) may not solicit as wide of a range of students’ ideas as do free response questions (38, 39). However, closed-ended questions can be useful in identifying the prevalence of incorrect conceptions (38). This type of information may be particularly useful for researchers and instructors who seek to characterize students’ ideas preinstruction to inform the design of curricula to target prevalent incorrect ideas. In this study, pairs of multiple-choice questions that address different facets of a similar topic (e.g., identification versus interpretation of a quantitative representation) are used to probe the depth of students’ understanding on the topic. Using pairs of questions at different difficulty levels can afford more opportunities to characterize students’ ideas.

### Study objective

This study aims to characterize students’ quantitative understanding of variation. Here, pairs of multiple-choice questions that query similar content are leveraged to achieve this objective. The pairs of questions focus specifically on two topics that are often challenging for students: descriptive statistics that represent variation, and interpretation of nonsignificant *p*-values (10, 14, 16, 25, 26, 30). In this study, responses from a large-scale administration of the pairs of questions are quantitatively analyzed to examine the types and patterns of ideas held by a large sample of students. Interviews are also conducted with a small sample of students who are asked to complete the same pairs of questions and describe their thought processes as they do so. The interviews are qualitatively analyzed to identify common themes in student thinking as it relates to these pairs of questions. The qualitative analyses of student interviews are used to support the conclusions from the large-scale question administration (47). This study aims to address the following research questions: (i) How do students reason about a mathematical expression of variation (descriptive statistics)? (ii) How does experimental context influence students’ interpretation of a nonsignificant *p*-value? (iii) How does prior statistics coursework affect students’ reasoning on multiple-choice questions about mathematical expressions of variation and interpretation of a nonsignificant *p*-value?

## METHODS

### Study context and participants

All students that were assessed in this study were enrolled in an introductory biology laboratory course at a large, Ph.D.-granting midwestern university in the Fall 2018 (*N_F18_* = 150) or Spring 2019 (*N_S19_* = 291) semesters. Students in this course are typically freshmen or sophomores. The student pool that is enrolled in these courses is 65% female and 35% male. Domestic students of color comprise 20% of the student pool. Approximately 20% of the student pool are first-generation college students. A total of 53% of participants (*N *=* *235) self-reported completion (or concurrent enrollment in) a statistics course in either high school or college. This study is approved under IRB STUDY00003137.

### Assessment administration

Students completed an assessment consisting of 16 multiple-choice questions on the first day of the semester as part of another study. The development and validation of this assessment (termed the Biological Variation in Experimental Design and Analysis [BioVEDA] assessment) is published elsewhere (48). Two of these questions that interrogated students’ ideas about interpreting *p*-values were already paired. An additional question was included to allow examination of students’ interpretations of descriptive statistics. This additional question underwent similar validation (e.g., expert review, student think-aloud interviews, and analysis according to classical test theory) as described in Hicks et al. (48). Students completed the assessment via Qualtrics during class time and were directed to choose the single best answer for each question. Question order was randomized. When taking the assessment, students could refuse permission to have their data used in the study. Of students across both semesters, 94% consented to have their data included in the study. Students from the Spring 2019 semester received 2 points of extra credit for completing the assessment. Students from the Fall 2018 semester did not receive extra credit for completing the assessment.

### Quantitative analysis of multiple-choice responses

To analyze differences in students’ understanding across pairs of questions, the percentage of correct responses were compared across question pairs using a chi-square test. A chi-square test was used to compare the frequency of answer choice selection across answer options. Differences in the frequency of answer choice selection between students who had and had not taken a prior statistics course were compared using a chi-square test. Results of statistical analyses were considered statistically significant at *p* < 0.05. Cramer’s *V* statistic is provided as a measurement of the effect size for chi-square tests.

### Student think-aloud interviews

As part of the development of the BioVEDA assessment (48), think-aloud interviews were conducted to examine the thought processes that students may use when answering the multiple-choice questions. Volunteer participants were recruited from an introductory biology lecture course via convenience sampling. An announcement was made during the class session to the entire class to invite students to participate in the interviews and an interview was completed with any student who agreed to do so. Rather than have every student answer all 20 BioVEDA questions, which would have been inordinately demanding of students’ time, students were randomly assigned to answer a set of four questions. Because the goal of the initial interview was question validation, five students were deemed sufficient to surface common difficulties. Student volunteers were not asked about any personal characteristics (e.g., demographic factors or prior statistics coursework). Five students were interviewed about the paired multiple-choice questions discussed in this study. Students are referred to by gender-ambiguous pseudonyms in the analyses below. Each participant was presented with the four questions described here (in sequential order) and asked to read the question and describe their thought process out loud as they decided which answer choice was best. If the student did not state why they chose (or did not choose) each answer option, they were prompted to do so by the interviewer. The interviewer was a graduate student pursuing a Ph.D. in STEM education who was not involved with developing the assessment questions or the conceptual design of this study. Each student completed all four questions, except for one (pseudonym Cameron), who did not complete the question shown in Fig. 2B due to time constraints. The think-aloud interviews were audio recorded following verbal consent of participants.

### Qualitative analysis of think-aloud interviews

Audio recordings of think-aloud interviews were deidentified, transcribed, and subsequently reviewed by two members of the research team who had not conducted the interviews. The first reviewer has a Ph.D. in molecular biology and has completed 2 years of postdoctoral training in education research and was involved in the development of the assessment questions. The second reviewer is a research assistant with a B.Sc. in biology who did not develop the assessment questions. Both reviewers independently described the processes students used to solve each problem and developed preliminary codes via inductive thematic coding (49, 50). Descriptions and associated preliminary codes from the two reviewers were compared to determine consensus, and any disagreements were resolved by discussion (51). Upon reaching consensus, the descriptions and associated preliminary codes were discussed and synthesized to identify common themes or strategies used by students in answering the question (50). Common themes or strategies used by students are described below.

## RESULTS

Analyses of two question pairs are presented below. The first question pair assesses students’ understanding of a mathematical expression of variation (the average absolute deviation from the mean). The second question pair assesses students’ understanding of a nonsignificant *p*-value in two experimental contexts (a small and a large sample size). For both question pairs, we first present quantitative analyses of data from the large-scale administration of these questions to identify generalizable patterns across a large number of student responses. We then describe qualitative analyses of students’ think-aloud interviews to explore the thought processes used by students to reason about the concepts in the question pairs.

### Many students can identify a mathematical expression of variation, but few can interpret its meaning

**FIG 1:**
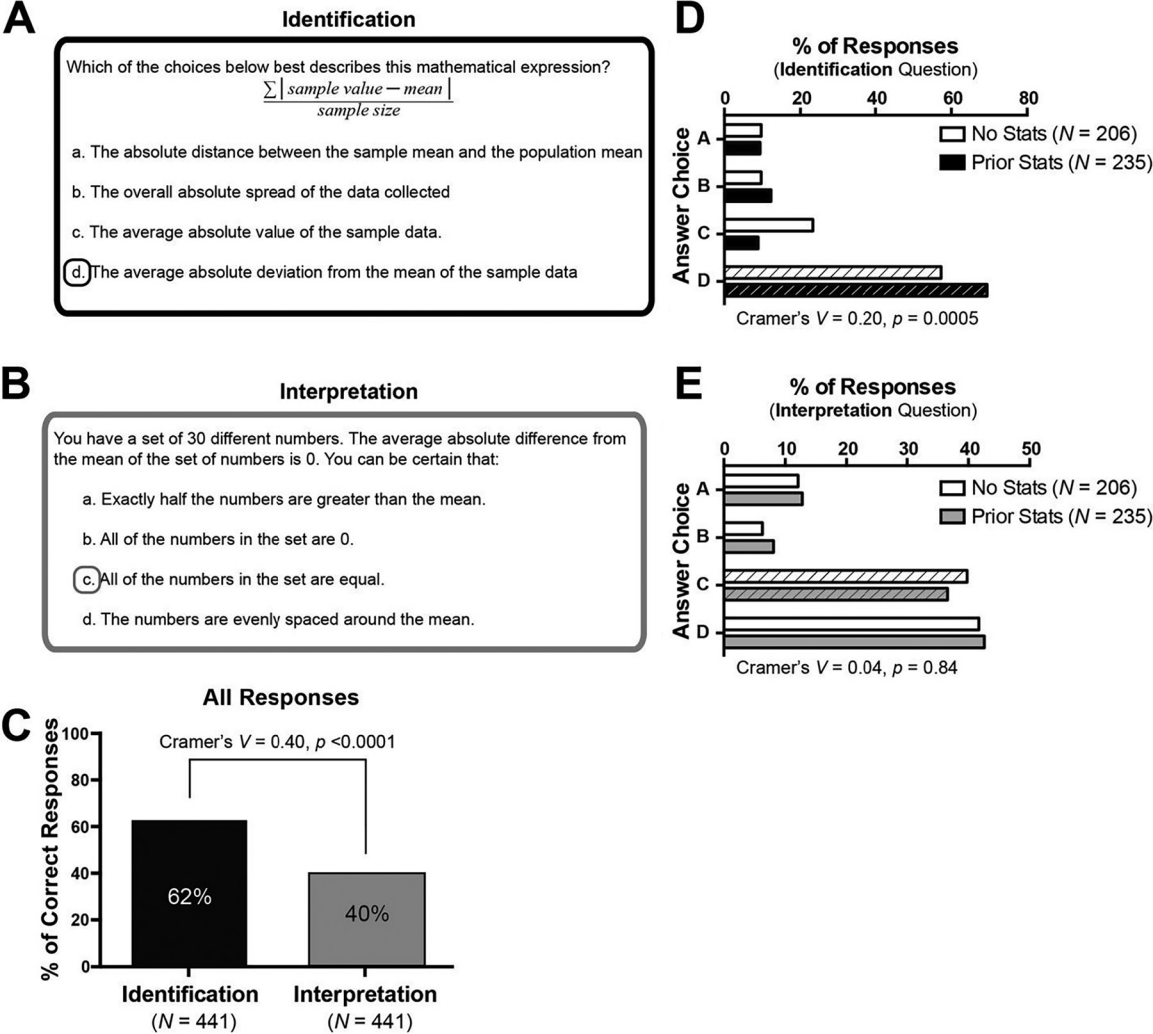
More students can identify a mathematical expression of variation than can interpret its meaning. (A) Question stem and answer options for the identification of a mathematical expression of variation. (B) Question stem and answer options for the interpretation of a mathematical expression of variation. (C) Percentage of students who correctly answered each item. (D) Percentage of students that chose each answer option for the identification question shown in panel A, disaggregated by prior statistics coursework. White bars indicate no prior statistics coursework, and black bars indicate prior statistics coursework. Best choice answer is indicated by diagonal lines on the bars. (E) Percentage of students that chose each answer option for the interpretation question shown in panel B, disaggregated by prior statistics coursework. White bars indicate no prior statistics coursework, and gray bars indicate prior statistics coursework. A best choice answer is indicated by diagonal lines on the bars. Cramer’s *V* statistic is provided to indicate effect size and *p*-values were determined via chi-square tests.

Representing variation in a data set is an important part of data analysis. Descriptive statistics, such as the standard deviation or variance, are one way to represent variation. Students who have been instructed on methods to calculate descriptive statistics for a data set may prioritize procedural thinking about these statistics over the conceptual ideas that they represent (19, 20). Thus, students were asked two questions testing their understanding of a mathematical expression of variation. One question asks students to identify the expression (Fig. 1A), and one asks students to interpret the expression (Fig. 1B). The mathematical expression for standard deviation was not used because (i) students may have received prior procedure- or calculation-focused instruction on this concept which could influence their thinking and (ii) students may have difficulty reasoning about this expression without prior exposure. Instead, the expression for the average absolute deviation from the mean was used since most students are likely naive to this expression. Conceptually, these mathematical expressions represent the same idea (52): in a given data set, how far away is any individual data point from the center?

### Quantitative analyses of responses from large-scale administration

More students are able to correctly identify the mathematical expression of variation than are able to interpret this expression [χ^2^(1) = 171.8, *p* < 0.0001, Cramer’s *V *=* *0.40] (Fig. 1C). While 62% of students can identify the math expression as the average absolute deviation from the mean, only 40% of students select the correct answer when asked to interpret what that expression means.

Student prior statistics coursework may influence their ability to identify and interpret mathematical expressions of variation (20). To determine the degree to which prior statistics coursework relates to students’ understanding of mathematical expressions of variation, the percentages of students that selected each answer option were disaggregated by students’ completion of a prior statistics course. A higher percentage of students with prior statistics coursework (69%) select the correct answer on the math expression identification question than do students who have not taken a statistics course [57%; χ^2^(3) = 17.7, Cramer’s *V *=* *0.20, *p* = 0.0005; Fig. 1D]. A higher percentage of students who have not taken a statistics course (23%) select distractor answer option C (*the average absolute value of the sample data*) than do students who have taken a statistics course (9%; Fig. 1D). Interestingly, students responded similarly to the question that asked for interpretation of the mathematical expression of variation, regardless of prior statistics coursework. Differences between percentage of responses from students who have and have not taken statistics on the interpretation question are neither sizable nor statistically significant [χ^2^(3) = 0.8, Cramer’s *V* = 0.04, *p* = 0.84; Fig. 1E].

### Qualitative analyses of student think-aloud interviews

Think-aloud interviews were conducted with student volunteers to examine how students reason through the identification and interpretation questions in Fig. 1A and B. Students participating in the interviews were presented with the identification and interpretation questions sequentially, unlike in the Qualtrics assessment where question order was randomized so these two questions were unlikely to be presented in this order. Many students employed similar approaches to reason through the identification question (Fig. 1A and Table 1). Four of five students selected the best choice answer as their correct answer. Students made sense of the mathematical concepts presented in the answer choices by attending to mathematical formalisms (i.e., operators, variables, and structure) within the symbolic expression provided in the question stem. The two prominent strategies used by students to make sense of the mathematical concepts by attending to mathematical formalisms were coordinating mathematical procedures with mathematical concepts and identifying operators and variables (Table 1). Only some students used the third strategy, relating to prior knowledge of standard deviation, to make sense of specific mathematical formalisms in the provided equation. These three strategies are discussed below.

**TABLE 1:** Common strategies used by students to answer the identification question shown in Fig. 1A

Strategy	Definition of strategy	Exemplar quote	Taylor	Jordan	Dana	Riley	Cameron
Coordinating mathematical procedures with mathematical concepts	Linking the mathematical procedure for calculating a quantity with the concept that the quantity represents	“…we’re dividing by the sample size, so that should be an average.”	Yes	Yes	Yes	Yes	Yes
Identifying operators and variables	Identifying or listing the operators and variables within the provided equation without verbalizing any interpretation of these elements	“Sample value minus mean divided by the sample size. We’re taking the sum of that called the absolute sum…”	Yes	Yes	Yes	Yes	Yes
Relating to prior knowledge of standard deviation	Connecting an unknown concept or mathematical expression to a previously learned concept or mathematical expression of standard deviation	“I would choose… the average absolute deviation from the mean of the sample data, because I know that [the numerator of the equation] is closely related to the standard deviation in a way.”	No	Yes	No	Yes	Yes
Selected correct answer			Yes	Yes	Yes	Yes	No

All five students coordinated mathematical procedures with mathematical concepts to make sense of the question (Table 1). Several students applied this strategy to reason about the concept of “average,” a word that was present in two of the answer options. Four students connected the mathematical procedure of “dividing by the sample size” to the mathematical concept of “average.” Some students, like Dana, used this reasoning to rule out options A and B, which don’t contain the word “average”: “So I see that it’s—we’re dividing by the sample size, so that should be an average. So that limits it to C and D.”

Identifying operators and variables was used at some point by all five students and was the first strategy used by three of the five students (Table 1). This strategy presented as a literal translation of the mathematical equation presented in the question stem into sentence format. Students did not always verbally identify each element in the equation. For example, Riley read the equation as “sample value minus mean over sample size.” Riley identified all of the variables within the provided equation (sample value, mean, and sample size) but only two of the operators (subtraction and division). Riley did not identify the summation or absolute value operators.

Three students related parts of the question to their prior knowledge of standard deviation to reason through the question. Two of these students applied their prior knowledge of standard deviation to recognize that the provided equation was similar to the equation for standard deviation. These students used this reasoning to justify selecting D (the correct answer: *the average deviation from the mean of the sample data*) as the best answer. Riley provided an example of this line of reasoning: “So I would choose… the [D] average absolute deviation from the mean of the sample data, because I know that [the numerator] is closely related to the standard deviation in a way.”

The third student, Cameron, also invoked their prior knowledge of standard deviation but did so to justify eliminating answer D: “…and with deviation, I feel like they mean by standard deviation and I’ve seen an equation for it before, and I don’t think it was that, so that’s the only reason I’m not choosing D.” Unlike the other students that drew on their prior knowledge of standard deviation, Cameron is more attentive to the differences than the similarities between the provided equation and the equation for standard deviation. Cameron is also the only student who selected an incorrect answer. None of the three students who referenced their prior knowledge of standard deviation specified exactly how the provided equation was similar to or different from the equation for standard deviation.

**FIG 2:**
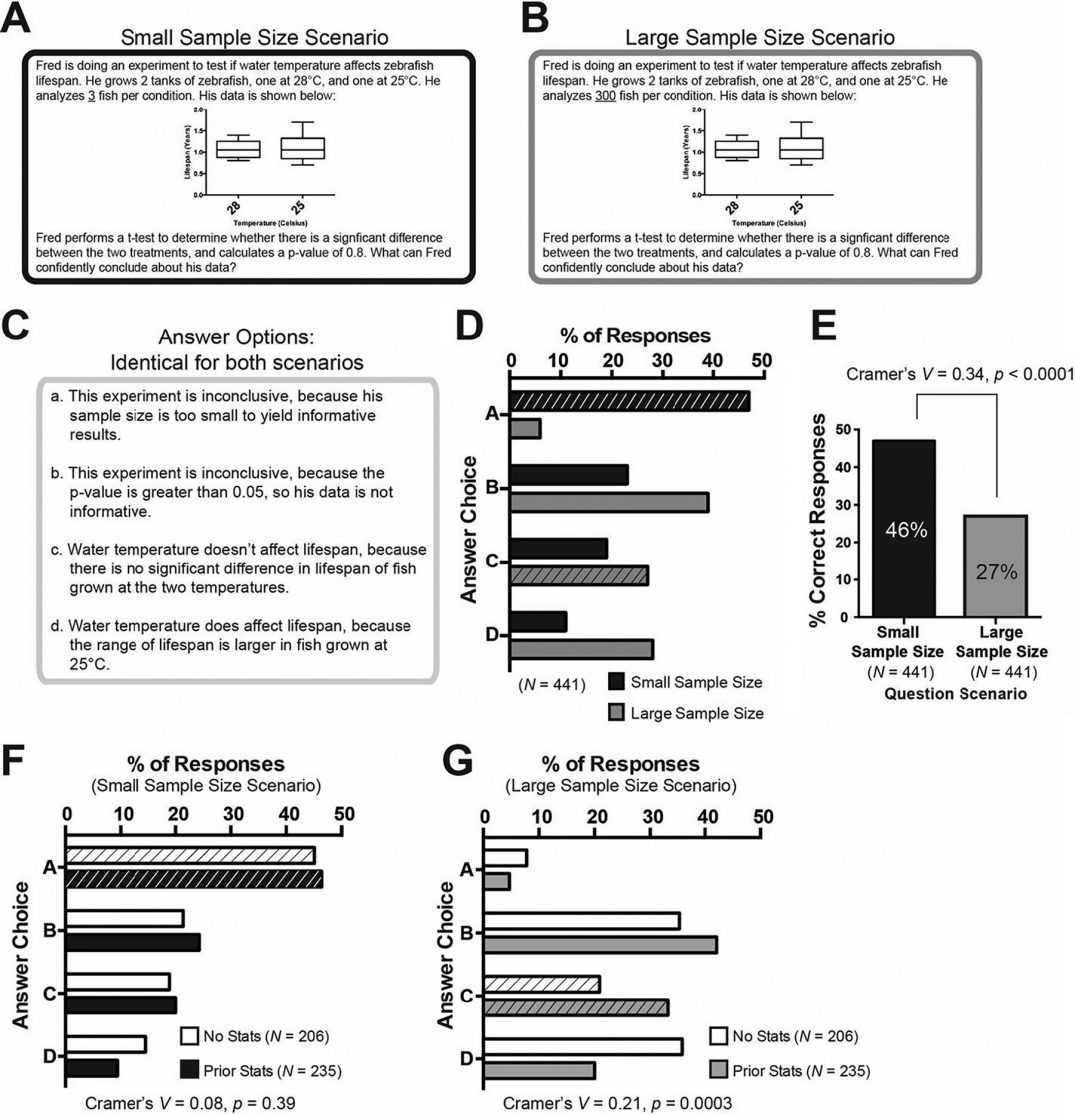
Many students fail to interpret a nonsignificant *p*-value as informative. (A) Question stem for the small sample size scenario. (B) Question stem for the large sample size scenario. (C) Answer options were identical for both scenarios. (D) Frequency that each answer option was chosen. The frequency for a small sample size scenario is shown in white bars, and the frequency for a large sample size scenario is shown in gray bars. A best choice answer is indicated by diagonal lines on the bars. (E) Percentage of correct responses on both question versions. (F) Percentage of students that chose each answer option for the small sample size question shown in panel A, disaggregated by prior statistics coursework. White bars indicate no prior statistics coursework, and black bars indicate prior statistics coursework. A best choice answer is indicated by diagonal lines on the bars. (G) Percentage of students that chose each answer option for the small sample size question shown in panel B, disaggregated by prior statistics coursework. White bars indicate no prior statistics coursework, and gray bars indicate prior statistics coursework. Best choice answer is indicated by diagonal lines on the bars. Cramer’s *V* statistic is provided to indicate effect size, and *p*-values were determined via Chi-square tests.

Students used a range of approaches to reason through the interpretation question (Fig. 1B and Table 2). Compared to the identification question, fewer students answered this question correctly (one of five students answered the question correctly). This is similar to the large-scale administration of this question (the majority of students chose an incorrect answer for this question; Fig. 2C).

**TABLE 2:** Common strategies used by students to answer the interpretation question shown in Fig. 1B

Strategy	Definition of strategy	Exemplar quote	Taylor	Jordan	Dana	Riley	Cameron
Coordinating mathematical procedures with mathematical concepts	Linking the mathematical procedure for calculating a quantity with the concept that the quantity represents	“The average absolute difference from the mean of this set is zero, that means that the average of all the numbers minus the mean is equal to zero.”	Yes	Yes	No	No	No
Relate to the idea of distribution	Rephrase or interpret the “difference from the mean” as an indicator of the distribution, balance, or normality of the data set	“*And the numbers are evenly spaced around the mean*. I think this would be the answer because if you have the mean and if the numbers are evenly spaced, that means one side has an equal amt of no. that are spaced same as the other side, which would give a difference. Because this would be positive and this would be negative.”	No	Yes	Yes	Yes	Yes
Relate to real-world knowledge	Connecting an unknown concept to the students’ knowledge of the real world to try to make sense of the concept.	“See, I wouldn’t do *all the no. in the set are equal [C]* just because they wouldn’t—I mean, they wouldn’t be equal if—I don’t really know about that question… It just doesn’t sound—it doesn’t make sense.”	No	Yes	No	Yes	No
Selected correct answer			Yes	No	No	No	No

A strategy used by two of the five students was coordinating the concept of the “average absolute difference from the mean” with a mathematical procedure, which required linking of the mathematical concept to mathematical formalisms. This differs from students’ approaches to the identification question, in which all students made connections between mathematical formalisms and mathematical concepts. Taylor (the only student to select the correct answer) accurately connected the concept with the mathematical procedures one would employ to calculate the average absolute difference from the mean: “… all the distances added together and divided by the sample size is equal to zero.” Jordan also rephrased the concept as a procedure but did so inaccurately, rephrasing the average absolute difference from the mean as “the average of all the numbers minus the mean.”

All four of the students who selected an incorrect answer related the concept of the “average absolute difference from the mean” to the idea of the distribution of the data and used this to justify choosing answer choice D (*all the numbers are evenly spaced around the mean*). Linking “average absolute difference from the mean” to the distribution of data is not inherently incorrect, since measures of deviation from the mean are commonly used to describe the distribution of a data set. However, students in this sample incorrectly interpreted an average absolute difference from the mean of 0 as an indicator of normality or balance within the data set. The reasoning behind this interpretation appears variable. For example, Jordan seems to conflate the concepts of median and average: “That means that the only way that [the average of all the numbers minus the mean = 0] could be possible is that the mean is the average. I mean, it has a perfectly shaped bell curve.”

Dana does not explicitly mention normality or a method of calculating the value of 0. Instead, Dana interprets this concept as an indicator of balance or symmetry in the data set: “half of the 30 would have to be above the mean, and half of the numbers—the rest of the numbers would be below the mean so that—and it’s evenly spaced out so that the set of numbers is zero.” Dana seems to be interpreting “the average absolute difference from the mean” as the sum of the directional distance of each value from the mean: values above the mean would be canceled out by values below the mean. This interpretation implies that Dana is not focusing on the word “absolute” in the question stem.

Two students applied their knowledge of the real world to try to make sense of this question. Jordan used this strategy to eliminate answer A: “We can’t be sure about that since there might be a slight deviation.” Jordan seems to be invoking knowledge that real life is rarely “exact” and says that they prefer answer D “for this whole reason that we can’t know if exactly half the numbers are greater than the mean.” Riley used their knowledge of the real world to eliminate answer C: “See, I wouldn’t do *all the numbers in the set are equal [C]* just because they wouldn’t—I mean, they wouldn’t be equal. […] It just doesn’t sound – it doesn’t make sense.” Riley seems to be verbalizing that all of the numbers being equal is not sensible but does not provide a clear rationale to explain why they hold this belief.

### Many students fail to interpret a nonsignificant *p*-value as informative

Statistical tests are often used to analyze biological data. The output of these tests, typically in the form of a *p*-value, must be interpreted to make inferences about the biological phenomenon being observed. Students were asked two questions that focused on the interpretation of statistical tests of biological data. Both questions presented a scenario in which a researcher has analyzed data and calculated a nonsignificant *p*-value of 0.8. In the first question, the researcher’s sample size is small (*N *=* *3) (Fig. 2A and C). In the second question, the researcher’s sample size is large (*N *=* *300) (Fig. 2B and C). The answer options were the same for both questions.

### Quantitative analyses of responses from large-scale administration

When students were presented with the small sample size scenario, 46% of students select the best answer that “the experiment is inconclusive, because his sample size is too small to yield informative results” (answer A; Fig. 2C to E). More students correctly interpret a nonsignificant *p*-value in the small sample size context than in the large sample size context [χ^2^(1) = 60.0, Cramer’s *V *=* *0.34, *p* < 0.0001, Fig. 2E]. When presented with the large sample size scenario, students shift away from this answer option, correctly recognizing that the sample is not too small to yield informative results. However, the most frequently chosen answer for the large sample size question [selected by 39% of students; χ^2^(2) = 99.5, Cramer’s *V *=* *0.27, *p* < 0.0001] was distractor B: *this experiment is inconclusive*, *because the p*-*value* *is greater than 0.05*, *so his data are not informative* (Fig. 2C and D). More students chose distractor B on the large sample size question than on the small sample size question [χ^2^(1) = 59.9, Cramer’s *V *=* *0.33, *p* < 0.0001; Fig. 2D]. 27% of students selected the correct response on the large sample size question: *water temperature doesn’t affect* life span, *because there is no significant difference in* life span *of fish grown at the two temperatures* (answer C; Fig. 2C to E). A similar proportion of students (27%) selected the incorrect response on the large sample size question stating: *water temperature does affect* life span, *because the range of* life span *is larger in fish grown at 25°C* (answer D, Fig. 2C and D).

Student prior statistics coursework appears to influence their reasoning about nonsignificant *p*-values, specifically on the large sample size question (Fig. 2F and G). The percentages of students who chose each answer for the small sample size question (Fig. 2A) are similar, regardless of prior statistics preparation [χ^2^(3) = 3.0, Cramer’s *V *=* *0.08, *p* = 0.39; Fig. 2F]. The percentages of students who chose each answer for the large sample size question (Fig. 2B) differ between students who have and have not had prior statistics coursework [χ^2^(3) = 19.2, Cramer’s *V *=* *0.21, *p* = 0.0003; Fig. 2G]. Most students (with or without prior statistics coursework) correctly determine that a sample size of 300 is sufficiently large, and few students select choice A (Fig. 2G). Students with prior statistics coursework select the correct answer (choice C) more frequently than do students without prior statistics coursework (Fig. 2G). Interestingly, students seem to favor different distractor options, depending on whether they have or have not taken a prior statistics course. Of students without prior statistics coursework, 36% select choice D compared to 20% of students with prior statistics coursework (Fig. 2G). Choice D states “*Water temperature does affect* life span, *because the range of* life span *is larger in fish grown at 25°C.*” Conversely, 42% of students with prior statistics coursework select choice B (*this experiment is inconclusive*, *because the p*-*value* *is greater than 0.05*, *so his data are not informative*), while 35% of students without prior statistics coursework choose this answer (Fig. 2G).

### Qualitative analyses of student think-aloud interviews

Think-aloud interviews were conducted with student volunteers (*N *=* *5) to identify thought processes that students used to reason about a nonsignificant *p*-value in both small (Fig. 2A) and large (Fig. 2B) sample size contexts. Students employed similar approaches to reason through both questions (Table 3). Four of five students selected the best choice answer as their correct answer on the small sample size question. None of the four students selected the best choice answer as their correct answer for the large sample size question (Cameron did not complete this question due to time constraints). Students answered these questions sequentially in the think-aloud interviews. This differs from the large-scale administration of the assessment, in which questions are randomized so students are unlikely to answer similar questions sequentially.

**TABLE 3:** Common strategies used by students to answer the small sample size question and the large sample size question

Strategy	Definition of strategy	Exemplar quote	Question version^*a*^	Taylor	Jordan	Dana	Riley	Cameron
Binary interpretation of *p*-value cutoff of 0.05	Recalls prior knowledge of *p*-value cutoff to inform a two-option interpretation	“So anything that has a *p*-value greater than, I think, 0.05 cannot be considered significant.”	Small	Yes	Yes	Yes	Yes	Yes
			Large	Yes	Yes	Yes	Yes	ND^*b*^
Application of previously learned ideas about optimal sample size	Using prior knowledge to make judgement calls about the appropriateness of a sample size	“…a sample size of three per condition is pretty small because then you can have random variation, and that could affect the results.”	Small	Yes	Yes	No	Yes	Yes
			Large	Yes	Yes	No	Yes	ND
Selected correct answer			Small	Yes	Yes	Yes	No	Yes
			Large	No	No	No	No	ND

a Small, small sample size question shown in Fig. 2A; Large, large sample size question shown in Fig. 2B.

b ND, no data (Cameron did not answer the large sample size question).

Four of five students volunteered an explanation of a *p*-value that included a reference to a cutoff value of 0.05 *before* reading the answer choices on the small sample size question (Cameron did so after reading choice B). All students referenced a binary interpretation of a *p*-value: if *p* > 0.05, conclude X, and if *p* < 0.05, conclude Y. Taylor linked this cutoff to significance: “So anything that has a *p*-value greater than, I think, 0.05 cannot be considered significant.” Dana stated that “*p*-values greater than 0.05 are inconclusive,” linking the cutoff to whether conclusions can be drawn from the data. Riley expressed both of these ideas, seemingly equating the concept of “significance” with being able to draw conclusions: “I think if [the *p*-value] is below 0.05, that it is significant, and if it’s above, that would mean you can’t really conclude anything.” Before selecting the correct answer for the small sample size question, students that declared a *p*-value greater than 0.05 as “inconclusive” (Jordan, Dana, and Riley) originally gravitated toward choice B that reads “*The experiment is inconclusive because the p*-*value* *is greater than 0.05 so his data are not informative*.” This choice aligned almost exactly with phrasing that students volunteered upon reading the term “*p*-value.”

Students referenced the ideas surfaced while answering the small sample size question to reason through the large sample size question. Students also compared and contrasted the two versions of the question while reasoning through the large sample size question. All four students referred to the binary interpretation of *p*-values that they initially verbalized and used this reasoning to identify the two answer choices (A and B) that said the “experiment is inconclusive” as top choices. All students selected the same distractor (choice B) as the best answer for the large sample size question: “*The experiment is inconclusive because the p*-*value* *is greater than 0.05 so his data are not informative*.”

Some students used their understanding of “optimal” sample size to reason about both question versions. Two students provided a conceptual basis for why a small sample size was problematic, and one of these students provided a rule for determining an “optimal” sample size. One student provided only a rule, and two students noted that the sample size was problematic without providing a supporting statement. When answering the small sample size question, Taylor and Cameron readily identified that *N *=* *3 is indeed small. Both students connected the idea of a larger sample size to more robust experimental analyses using non-technical language. Taylor seems to connect the concept of sample size to an informal understanding of statistical power: “…because you need a larger sample size to even run a good *t* test.” Cameron noted that a small sample size is not optimal because “then you can have random variation, and that could affect the results.” Two students mentioned previously memorized rules about determining a “good” sample size, referencing specific number cutoffs. Taylor said “I think the sample size cutoff is 30 or greater, I think. And so if it’s below 30, the sample size too small to run a good *t* test, but if it’s above 30, then it’s good to run a *t* test.” Jordan also invoked a rule for determining an “optimal” sample size, but unlike Taylor, does not provide any additional conceptual reasoning to justify why a small sample size is problematic. Jordan identifies *N *=* *10 as an optimal sample size: “We could argue that the sample size is too small. Indeed, the sample size should—if you’re doing *p*-value, this should be greater than 0.05, but then there’s only 3. Optimally, it’d be 10.” Riley and Dana also noted that *N *=* *3 is a small sample size, but they did not provide an explanation of why a small sample size is undesirable. Jordan and Dana did not select choice A (*The sample size is too small to yield informative results*) for the small sample size question until moving on to read the question stem for the large sample size question (where *N *=* *300). These students subsequently revised their answer selection and chose answer choice A (both had originally chosen choice B: *the experiment is inconclusive because the p*-*value* *is greater than 0.05*).

## DISCUSSION

Multiple-choice questions can provide valuable information about students’ ideas, but the information that can be gleaned from a student’s response on a single question is bounded by the answer options provided. Administering pairs of questions that focus on a single topic allows for a richer characterization of students’ understanding of the content. Here, think-aloud interviews were also conducted to examine the thought processes used by a small sample of students, and the interviews support conclusions drawn from analyzing responses from a large-scale administration of the same questions. The following sections discuss students’ ideas about conceptual material from two pairs of questions that focus on mathematical expressions of variation in a data set and interpreting the results of statistical tests.

### Students struggle with interpretation of statistical expressions

One of the pairs of questions discussed in this study probes students’ understanding of a mathematical expression of variation in a data set (Fig. 1). The paired questions focus on the mathematical expression for the average absolute deviation from the mean, an expression that represents how far an individual data point is, on average, from the mean of a data set. This expression uses more simplistic mathematic operations than does the expression for standard deviation, which represents a similar concept (52). Though students are unlikely to have encountered the average absolute deviation from the mean in previous coursework, the majority of students can reason through the symbolic expression and correctly identify the expression as the average absolute deviation from the mean (Fig. 1D and Table 1). The quantitative analysis of the multiple-choice data revealed that students who have had prior exposure to common summary statistics (e.g., standard deviation) through prior statistics courses may be better able to recognize the form of this symbolic expression as one that describes the spread of a data set (Fig. 1D). However, students who relied on their prior knowledge of standard deviation in the interviews did not always do so successfully (Table 1), suggesting that prior exposure to statistics concepts does not ensure conceptual understanding.

When asked to interpret what the mathematical expression represents (Fig. 1B), the majority of students choose an incorrect answer (Fig. 1C and Table 2). Prior statistics coursework does not appear to influence students’ ability to interpret the mathematical concept (Fig. 1E). Both the quantitative and qualitative analyses reveal that some students seem to be equating the concept of “average absolute difference from the mean” (a measure of data spread) with the concept of a balanced or symmetrical distribution (Fig. 1E and Table 2). Previous work has shown that students may default to a calculation-oriented view of descriptive statistics (as opposed to viewing these quantities as useful summary descriptors of a data set) if they have been previously taught these statistics in a manner that prioritizes correctly completing the computational procedure over understanding what the calculated values represent (19, 20, 27). The present study extends this work by focusing on a statistical expression that students are likely naive to and on which they would not have received instruction. Our data suggest that even in the absence of prior instruction that focused on a calculation-oriented use of a mathematical expression, many students have difficulty extracting meaning from mathematical representations.

While all five students who participated in think-aloud interviews were able to make connections between the mathematical concepts and mathematical procedures presented in the identification question (Fig. 1A and Table 1), only two students made connections between concepts and procedures for the interpretation question (Fig. 1B and Table 2). The only student who correctly coordinated the mathematical concept with a procedure was also the only interviewed student to choose the correct answer for the interpretation question. Students who can successfully coordinate the mathematical concept with the relevant mathematical structure and procedures may be better able to make sense of the concept (28, 29).

Two students also invoked their knowledge of the real world to make sense of the “average absolute difference from the mean,” but neither student did so correctly (Table 2). Both students seem to be expressing notions of what is (or is not) likely: a perfectly symmetrical distribution is unlikely, and all 30 numbers in the set are not likely to be equal. It is worth noting that the student volunteers were pulled from a biology course, so they may have been primed to think about this question using a biological lens. In biological systems, it is indeed true that variation in a measured outcome is far more likely than the complete absence of variation. However, the question does not imply a biological context, nor does the question specify how the 30 numbers in the set were selected. Students are often encouraged to leverage their real-world knowledge to make sense of mathematical concepts (53, 54). However, in this case, students’ application of their knowledge of the real world in this artificial context are preventing them from correctly making sense of the concept. Students may have been better able to appropriately apply their real-world knowledge in a scenario that was more realistic (i.e., in which variation was present).

### Some students perceive *p*-values to encode binary information: significant *p*-values are informative, while nonsignificant *p*-values are not

The second question pair presented in this study ask students to interpret the results of a statistical test that yielded a nonsignificant *p*-value (Fig. 2A to C). The two questions differ only in the sample size used to generate the data—a small sample size of 3 or a larger sample size of 300. Students have more difficulty interpreting the results in the large sample size scenario than in the small sample size scenario (Fig. 2E and Table 3).

Students were equally likely to decide that these data supported the correct conclusion (that water temperature *does not* affect life span) or the incorrect conclusion (that water temperature *does* affect life span, because the range of life span is larger in fish grown at 25°C). Approximately a quarter of students selected this incorrect distractor, suggesting that some students believe that any indication of differences between data sets (e.g., the range of data points) implies a causal relationship between the manipulated variable and the measured outcome. This distractor appears particularly attractive to students who have not taken a prior statistics course.

Interestingly, the idea that any difference between data sets is indicative of statistical significance is seemingly at odds with the most frequently chosen answer option for the large sample size scenario. This option reads “This experiment is inconclusive, because the *p*-value is greater than 0.05, so his data are not informative.” All students in our sample who completed think-aloud interviews on these questions expressed binary interpretations of *p*-values and used this line of thinking to justify selecting this answer option for the large sample size question. Together, these data indicate that some students may think of *p*-values as a binary indicator of data quality—only results with significant *p*-values should be trusted, whereas all other data are uninformative. This notion is consistent with previously documented alternative ideas that a nonsignificant *p*-value is evidence of a failed experiment or a result with no practical significance (32, 33, 35–37). This belief seems to be more firmly held by students who have taken a prior statistics course compared to students who have not (Fig. 2G), suggesting that statistics instruction may be reinforcing this type of binary thinking. A similar phenomenon has been hinted at by other studies, suggesting that incorrect ideas about *p*-values may be particularly difficult to shift (31–33, 36).

### Students use rule- or procedure-based approaches to think quantitatively about variation

Both of the question pairs discussed above ask students to apply a quantitative understanding of variation to identify the correct answer choice. In both question pairs, many students have an incomplete understanding of the concept being assessed; the majority of students can identify the symbolic expression for the average absolute deviation from the mean, and many students correctly interpret a nonsignificant *p*-value in the context of a small sample size. However, when the question is made more challenging by asking for interpretation of the identified symbolic expression, or by changing the context to a large sample size, students’ incomplete understanding begins to break down, and fewer students select the correct answer. This may be due to a focus on procedures and memorized rules to the exclusion of a more nuanced understanding of the underlying concepts. Many students were able to identify the procedures encoded in the symbolic mathematical notation to recognize the mathematical representation of variation, but fewer students correctly understood what that quantity represented. When reasoning about the interpretation question during think-aloud interviews, most students did not make accurate connections between mathematical procedures and concepts (a strategy that had helped students make sense of the identification question) and instead relied on incorrect conceptual knowledge of tangentially related statistical concepts or of the real world. Several studies have reported student difficulties with linking symbolic mathematical expressions with the underlying phenomena that they represent in multiple disciplines (19, 20, 22–26).

This type of incomplete understanding also manifests itself when students have to make judgement calls in light of statistical test results. When students are asked to make a conclusion about a relatively straightforward scenario (small sample size, nonsignificant *p*-value), many students select the appropriate answer, which states that no conclusions can be made because the experimental design is flawed (the sample size is too small). In this scenario, it is plausible that the large *p*-value is due to a dearth of evidence, and it is difficult to conclude anything substantial about the underlying biological phenomenon based on observations from just three individuals. Students noted this in think-aloud interviews, stating that the small sample size was not ideal. In some cases, students provided a conceptual rationale to justify their assertion that a small sample size is not optimal. In other cases, students vaguely dismissed the sample size as being suboptimal without a conceptual justification for this assertion or invoked previously memorized rules to judge whether the sample size was adequate. When the scenario is changed (large sample size, nonsignificant *p*-value), many students apply this same binary logic: if the *p*-value is large, nothing can be concluded about the phenomenon being studied. This almost Boolean thought process leads to an overly simplistic understanding of statistical test results that can preclude students who think in this manner from making accurate conclusions about biological data.

### Implications for teaching

This and other studies indicate that prior instruction does not always result in correct conceptual understanding of variation (19, 20, 27). Our findings suggest that students who can successfully coordinate the mathematical concept of variation with the relevant mathematical structure and procedures may be better able to make sense of the concept (28, 29). Instruction that presents calculating mathematical expressions of variability in data sets as routine procedures that must be carried out as part of data analysis may push students to prioritize this type of calculation-oriented or procedural view of mathematical expressions over deep conceptual understanding. Adjusting instruction such that students must coordinate mathematical procedures with the concepts that they represent (e.g., by inventing their own mathematical procedures to represent a statistical concept) may be one strategy to foster deeper conceptual understanding (55–58).

Our findings suggest many students are largely unclear about what can be confidently concluded from a nonsignificant *p*-value, and that prior statistics instruction may be reinforcing incorrect ideas about *p*-values. Instruction that places less emphasis on binary decision-making using the *p*-value and instead emphasizes how to assess the appropriateness of an experimental design, the presence and magnitude of differences between experimental conditions, and the significance and meaningfulness of the outcomes of statistical tests may help students strengthen their ability to interpret statistical tests of biological data. Other studies have indicated that many instructors also hold incorrect ideas about *p*-values (32). Thus, it may also be beneficial to provide professional development to support instructors’ conceptual understanding and ability to facilitate lessons in which students critically assess statistical test results in light of multiple facets of experimental design.

### Study limitations

This study of student ideas is limited by the answer options provided in the questions (59). The analysis presented here is not intended to be an exhaustive characterization of students’ ideas or understanding of representations of variability or interpretation of statistical results. However, our data point to interesting patterns of students’ ideas that merit deeper exploration. In-depth, open-ended student interviews or constructed response questions that elicit a broader range of responses could be used to further probe and characterize students’ ideas and suggest pathways for instructional interventions to enhance student cognition.

Our qualitative analysis of the ideas that students expressed during the interviews are also limited by the number of participants. Including a greater number of students may result in a wider range of ideas expressed than we were able to capture in this study. We also did not ask students about their prior statistics or biology laboratory coursework, although some students volunteered this information during their interview. Including interview questions about prior academic preparation may be useful in drawing conclusions about the extent to which students’ coursework may have influenced their thinking.

### Conclusions

Many students have difficulty thinking quantitatively about variation in the context of a biological investigation. It is important to characterize students’ ideas about variation in biological investigations in order to develop curriculum that elevates students’ understanding of key topics that prove difficult. This study provides two examples of how pairs of multiple-choice questions that query a similar topic can be used to provide deeper insight into student thinking than single multiple-choice questions alone, and with greater ease than written response questions. This type of assessment design may be a useful technique that instructors or researchers can use to quickly evaluate the effectiveness of instruction or to characterize student ideas. Quantitative and qualitative analysis of student responses on two question pairs indicate many undergraduate introductory-level biology students apply procedure-focused or rule-based thinking processes to reason about descriptive statistics and statistical tests. These data, together with previously published work, suggest students may benefit from additional instruction focused on conceptual understanding of difficult topics like mathematical expressions of variation (e.g., standard deviation and variance) and interpreting statistical tests (e.g., *t* tests) and resulting *p*-values (6, 14, 16, 19, 20).
